# Coma Due to Malaria

**Published:** 1910-05-21

**Authors:** 


### COMA DUE TO MALARIA.

The malignant type of malaria, in which coma often occurs, is fortunately not so common as it used to be even in the tropics, and it is decidedly rare in temperate countries. Nevertheless, it may occur occasionally even in Great Britain in people who have been subject to malaria when resident abroad. The following case may serve as an example of the difficulty that may arise in the diagnosis of such a case.

The patient was a man aged thirty-seven, who was found unconscious on August 10 at a railway terminus, whence he was forthwith transferred to a hospital. When seen there he was completely comatose with flaccid muscles and stertorous breathing; there were neither twitchings nor convulsions. Reflex movements were in abeyance. There was no evidence of facial paralysis and no conjugate deviation of either his head or his eyes. The knee-jerks and the tendo Achillis jerks were exaggerated, but there was no extensor plantar reflex on either side. The pupils were equally dilated, of normal shape, and they were inactive to light. There was no photophobia and no pain when the eyeballs were pressed. Urine and fæces were passed involuntarily. There was no œdema and no evidence of serous effusion. The colour was livid and the face was congested. The temperature was 104° F., the pulse rate 120 per minute, and the pulse soft and regular. No abnormal physical signs could be detected in the lungs or heart. The liver was of normal size, and the spleen could not be felt. The urine contained neither sugar nor albumin. The diagnosis was quite obscure, until a history was obtained that the man had resided for two years in the Belgian Congo, whence he had returned only a few days previously. Curiously enough, during his stay in Africa he had never suffered from any illness, and it was only whilst he was on the mail-boat returning home that he first presented any signs of paludism of the ordinary form with rigors, pyrexia, and sweating. He had been a temperate man, was not addicted to drink, and had never had syphilis. He was treated forthwith with 20 grains of sulphate of quinine in a mixture, an injection of 15 grains of mono-chloral hydrate each day for a week, spartein and camphorated oil. The day after admission he had recovered some degree of consciousness; for the next three days he remained in the same state of prostration, and the temperature remained up at about 104° F. without remission. Both urine and fæces were passed involuntarily, the former being small in amount and very dark coloured. The skin was dry. There were no pains, but only a sense of great fatigue; there was no headache. From this time forward steady progress towards recovery was made, the temperature falling gradually to normal. The exaggeration of the reflexes disappeared and on August 23 the man was discharged at his own request.

### Points in Diagnosis.

A blood examination was made and also an investigation of the fluid obtained by lumbar puncture. It should be mentioned that no malarial parasites were found, but the blood examination was only made after large doses of quinine had been given. It may be that the diagnosis of malarial coma was wrong; nevertheless, nothing more probable could be suggested to fit the case, the main features of which were complete coma with marked pyrexia in a patient who had recently returned from the tropics and who had suffered from paludism on board ship.

The high temperature, the absence of albuminuria, the normal rhythm of the respiration and of the heart serve to exclude the diagnosis of uræmia, and diabetic coma was out of the question. As regards a cerebral hæmorrhage, even if one might use as arguments in favour of this diagnosis the acute onset, the exaggeration of some of the reflexes, and the early and high pyrexia, there was against it the absence of the ordinary signs of hemiplegia, the absence of conjugate deviation, and the absence of extensor plantar reflex. Alcoholic coma could be excluded not only by the high pyrexia, but also by the absence of any alcoholic odour in the breath or of any evidence of drink.

On the other hand, when the patient was first seen it was much more difficult to exclude a diagnosis of that coma which sometimes terminates a case of tuberculous or cerebro-spinal meningitis that has hitherto been latent. One is familiar with ambulatory forms of this disease which may end almost like an apoplexy. There were, however, no convulsions, no twitchings, no paralysis, no Kernig's sign, no vomiting, and the termination of the disease so rapidly and favourably was against either of these diagnoses. It was chiefly on the history and upon the course that the diagnosis was made.

